# Psychometric Correlation of Personality Disorders Along With Defense Mechanisms in Mexican Individuals With Depressive and Anxious Disorders: A Cross-Sectional Study

**DOI:** 10.7759/cureus.33805

**Published:** 2023-01-15

**Authors:** Roxana Galván-Suárez, Martha Patricia Ontiveros-Uribe, Enrique Chavez-León

**Affiliations:** 1 Department of Teaching, National Institute of Psychiatry, Mexico City, MEX; 2 Department of Psychology, Anáhuac University, Mexico City, MEX

**Keywords:** personality theories, psychometric tests, personality tests, clinical anxiety, depression

## Abstract

Background

Personality disorders are a multi-theoretical construct that encompasses predictable and quantifiable behavioral, cognitive, and affective characteristics in individuals. Previous studies underscore the existence of a relationship between personality disorders and defense mechanisms, these being coping styles that arise unconsciously in the face of adversity and that have adaptive purposes. There is evidence that alludes to a connection between pathological personality, defense mechanisms, and their relationship with negative mental health outcomes, such as depressive and anxious symptoms. The objective of this study was to study, psychometrically measure, and associate personality disorders, defense mechanisms, and depressive and anxious symptoms.

Methodology

A cross-sectional study was conducted on 81 participants with major depressive disorder, generalized anxiety disorder, and panic disorder who received treatment at a tertiary care institution between July 2021 and February 2022. Psychometric instruments were employed to evaluate the study variables, such as the Beck Depression Inventory (BDI), the Hamilton Depression Rating Scale (HAM-D), the Hamilton Anxiety Rating Scale (HAM-A), the Personality Diagnostic Questionnaire - Version 4 (PDQ-4) Plus, and the 40-item Defensive Styles Questionnaire (DSQ-40).

Results

Depressive and anxious symptoms were related to the scores obtained in the Defensive Styles Questionnaire and the Personality Disorders Questionnaire. Some defense mechanisms were positive predictors of the score between these scales.

Conclusions

There is a relationship between personality traits and defense mechanisms that could influence the development and severity of depressive and anxious psychopathology in this population.

## Introduction

Background

Depressive disorders belong to a diverse category of psychiatric disorders that are characterized by a depressed or irritable affect, along with a series of internalized and externalized phenomena that lead to a loss of functionality in various areas of life [1]. They are one of the leading causes of the burden of disease and are often associated with anxiety disorders [2]. These represent a wide range of phenomena related to perseverative cognitions and recurrent avoidance of overt or perceived threats [1]. Regarding the first point, major depressive disorder (MDD) stands out as the most frequent diagnosis in the category of depressive disorders [3]. On the other hand, generalized anxiety disorder (GAD) and panic disorder (PD) are its most common comorbidities [4]. Collectively, personality disorders encompass a range of affective, behavioral, and cognitive styles that stably persist in a person and predict how that individual negatively responds to the environment; these disorders impact various areas of functionality, from the way one relates to others, to how one works or performs their daily activities [5]. Therefore, the decrease in psychosocial functioning that occurs in personality disorders can be understood as a possible mediator of the development of comorbid or future psychopathology [6]. Historically, defense mechanisms have been a pillar for understanding the severity of pathological personalities [7]. These represent internal or external actions resulting from intrapsychic conflicts and have an adaptive function; however, studying defense mechanisms is often a complex task, as they require training and time to diagnose in clinical and psychodynamic settings. For this reason, efforts have been made to measure them psychometrically on time [8]. Thus, their study has been less common since the cognitive science revolution, but their importance cannot be dismissed since they have an intrinsic relationship with personality development and their subsequent link with other psychiatric comorbidities [7]. That said, in recent times, there have been efforts by neuroscience to relate all these factors harmoniously [9]. For this reason, the objective of this study was to measure personality disorders and depressive and anxiety disorders, as well as defense mechanisms with psychometric instruments in a Mexican population, to find associations between the resulting data.

## Materials and methods

A cross-sectional study was conducted to determine the impact of factors of personality disorders on the defense mechanisms of individuals with MDD, GAD, and PD in the National Institute of Psychiatry, Mexico City, Mexico, from July 2021 to February 2022. A total of 81 subjects were included using nonprobability convenience sampling. Inclusion criteria encompassed male or female individuals aged 18 to 65 years being treated for MDD, GAD, or PD under the criteria of the Diagnostic and Statistical Manual of Mental Disorders - Fifth Edition (DSM 5) [1]. Concomitantly, subjects with any psychotic, bipolar, obsessive, impulse-control, neurocognitive, substance-use, or developmental disorder were excluded from the project. An interview was done to collect information about sociodemographic data. The diagnosis was assessed and based on a clinical interview and examination that used the diagnostic criteria of the DSM 5 intended for the formerly disclosed disorders. Personality disorders were assessed with the Personality Diagnostic Questionnaire - Version 4 (PDQ-4). PDQ-4 is a 100-item, self-administered, true-false questionnaire that yields diagnoses consistent with the DSM-IV diagnostic criteria for Axis II disorders [10]. Additionally, the 40-item Defense Style Questionnaire (DSQ-40) was used to measure defense mechanisms. DSQ-40 is a self-report inventory that assesses groups of defenses called defensive styles according to Vaillant’s continuum, ranging from immature or maladaptive defenses to mature ones. The 40-item version is a widely used self-report measure, given its easy administration and cost-effectiveness [11]. In equal measure, depressive symptoms were assessed with both the Hamilton Depression Rating Scale (HAM-D) and the Beck Depression Inventory (BDI). These validated tools feature items that determine elements such as the presence of depressive affect, anhedonia, and somatic symptoms, among others [12]. At the same time, the Hamilton Anxiety Rating Scale (HAM-A) was used to assess these clusters of symptoms in the same population [13]. Also, this study was carried out in accordance with the Declaration of Helsinki, no personal information was gathered, and answers were kept confidential. All participants provided written informed consent. The investigation was approved by the National Institute of Psychiatry’s Ethics Committee with certificate number CONBIOETICA-09-CEI-010-20170316. Data was analyzed with the software Statistical Package for Social Sciences, version 20 (IBM Corp., Armonk, NY, USA). Frequencies and percentages were calculated for qualitative variables. Means (M) and standard deviations (SDs) were computed for quantitative variables. Pearson correlation test was done to assess the relationship between personality traits with defense mechanisms and depressive and anxious symptoms. Additionally, a regression model was used to predict the influence of the variables of the study. Relative to this, *P*-value < 0.05 was deemed statistically significant.

## Results

Study findings are described by the diagnostic groups, beginning with sociodemographic data (Table 1), and followed by clinical characteristics and defense mechanism scores. In the end, the results of the correlations and multiple regression analysis are detailed.

**Table 1:** Sociodemographic characteristics by the diagnostic groups. MDD, major depressive disorder; PD, panic disorder; GAD, generalized anxiety disorder

	MDD	PD	GAD
n	27	27	27
Gender			
Women, *n* (%)	23 (85)	13 (48.1)	22 (81.5)
Men, *n* (%)	4 (15)	14 (51.9)	5 (18.5)
Age (years)	30.41 ± 12.42	36.48 ± 12.5	39.11 ± 13.53
Education (years)	11.83 ± 2.74	12.48 ± 3.24	12.56 ± 2.93
Activity			
Employed, *n* (%)	9 (33.33)	19 (70.4)	13 (48.1)
Students, *n* (%)	8 (29.63	2 (7.4)	3 (11.1)
Stay-at-home spouse, *n* (%)	4 (14.81)	3 (11.1)	5 (18.5)
Unemployed, *n* (%)	6 (22.22)	3 (11.1)	6 (22.2)

All participants completed the interventions relevant to the study. The mean age was 35.98 ± 12.97 years, and 35 participants (42.70%) had subsyndromic features of a personality disorder or did not integrate one.

Major depressive disorder

The group consisted of 27 participants with a diagnosis of MDD. Twenty-three were women alongside four men, with a mean age of 30.41 ± 12.42 years (age range from 20 to 59 years), mostly single (*n* = 16, 59%), with studies up to high school (*n* = 13, 48.2%), average schooling of 11.83 ± 2.74 years, and most doing some work or academic activity (*n *= 21, 77.8%).

Most had recurrent MDD (*n *= 22, 81.88%) and scored between very severe (*n *= 11, 40.74%) and moderate (*n *= 9, 33.33%) intensities on the HAM-D scale, with an average score of 20.44 ± 5.7 in this scale, 12.37 ± 8.74 in the HAM-A scale, and 21.41 ± 8.55 in the BDI scale. Of the 27 participants, 18 (66.7%) had a personality disorder, mainly borderline personality disorder and 9 (33.33%) did not integrate any. The frequency of personality disorders in participants with mild-moderate (58.3%) and severe-very severe (73.3%) depression was similar but lacked significance (chi-square 0.675, general linear model = 1, *P *= nonsignificant).

Also, this group was divided into participants with mild and moderate depression (*n *= 12) and those with severe and very severe depression (*n *= 15). Patients with mild-moderate depression had higher scores (7.2 ± 2.06) than participants with severe-very severe MDD (5.5 ± 2.11) in the rationalization defense mechanism (*t* = 2.55, df 25, *P *= 0.041). In contrast, participants with very severe depression had higher scores regarding somatization (7.33 ± 1.7 versus 4.75 ± 2.23; *t *= 3.43, df 25, and *P *= 0.002). No correlation was found between HAM-D or HAM-A scale scores with any defense mechanisms. In addition, the BDI score was negatively correlated with the defense mechanism of anticipation (*r *= -0.458, *P =* 0.16) and positively correlated with devaluation (*r *= 0.394, *P *= 0.042). Both defense mechanisms showed their weight in the BDI scores in the multiple regression analysis: anticipation (*β *= -0.428, *t* = 4.72, and *P *= 0.017) and devaluation (*β *= 0.358, *t* = 2.14, and *P* = 0.042).

Panic disorder

The group consisted of 27 participants, 48.1% being female, with an average age of 36.48 ± 12.5 years; 51.9% were single; up to 37% finished high school education; and also most of the individuals were laboring or doing an academic activity 88.9%.

The average score of these participants on the HAM-A scale was 24.89 ± 6.92; on the HAM-D scale, it was 13.26 ± 4.76; and on the BDI scale, it was 16.52 ± 10.12 points. This group was the one with the lowest frequency of personality disorders, a little over 40%. However, borderline personality disorder was the most frequent (14.8%). No difference was observed in the frequency of the diagnosis of personality disorder among the three groups (chi-square 30.87, general linear model = 2, and *P *= nonsignificant). Also, no significant differences were found in the scores of the HAM-D, HAM-A, and BDI in participants with a personality disorder versus those without it. The HAM-A score correlated only with the pseudo-altruism defense (*r *= 0.412 and *P* = 0.033) in the second group (individuals with PD). In the regression analysis, it was also significant (*β *= 0.412, *t* = 2.26, and *P* = 0.033). The HAM-D score also correlated with only one defense mechanism: sublimation (*r *= 0.410 and *P* = 0.033). In the regression analysis, it was also significant (*β*= 0.41, *t* = 2.25, and *P* = 0.033).

Regarding the defense mechanisms, it would seem that these had a proportional impact on the way in which the BDI was answered. The defenses that correlated significantly with the instrument were sublimation (*r *= 0.441 and *P *= 0.021), pseudo-altruism (*r *= 0.450 and *P* = 0.018), acting out/exo-acting (*r *= 0.416 and *P* = 0.031), isolation (*r *= 0.412 and *P* = 0.033), and displacement (*r *= 0.549 and *P* = 0.033). In the multiple regression analysis, both sublimation (*β *= 0.429, *t* = 2.93, and *P* = 0.007) and displacement (*β *= 0.540, *t* = 3.69, and *P* = 0.001) significantly influenced the BDI score.

The relationship and weight that defense mechanisms had with the severity of depression and anxiety were determined through Pearson's correlation coefficient (*r*) and multiple regression analysis (*β*). Pearson's correlation was used to identify the defense mechanisms that could influence the scores (severity) of the depression and anxiety scales. With the multiple regression analysis, the defense mechanisms that had a significant impact on the scores of the depression and anxiety scales were determined. The rest of the clinical characteristics of the three groups are shown in Table 2.

**Table 2:** Clinical characteristics of the diagnostic groups (Hamilton scales and PDQ-4). PDQ-4, Personality Diagnostic Questionnaire - Version 4; HAM-D, Hamilton Depression Rating Scale; HAM-A, Hamilton Anxiety Rating Scale; MDD, major depressive disorder; PD, panic disorder; GAD, generalized anxiety disorder

	MDD	PD	GAD
n	27	27	27
HAM-D	20.44 ± 5.70	13.26 ± 4.76	9.33 ± 5.06
HAM-A	12.37 ± 8.74	24.84 ± 6.92	13.48 ± 6.82
BDI	21.41 ± 8.55	16.52 ± 10.12	17.07 ± 11.74
Personality disorders	18 (66.67 %)	11 (40.7%)	17 (63%)
Borderline	12 (66.6%)	4 (14.8%)	6 (22.2%)
Narcissist	2 (11.11%)	1 (3.7%)	2 (7.4%)
Schizotypal	1 (5.55%)	-	-
Obsessive-compulsive	1 (5.55%)	2 (7.4%)	-
Dependent	1 (5.55%)	1 (3.7%)	2 (7.4%)
Depressive	1 (5.55%)	-	-
Schizoid	-	1 (3.7%)	1 (3.7%)
Avoidant	-	2 (7.4%)	6 (22.2%)
No personality disorder	9 (33.33%)	16 (59.3%)	10 (37%)

Generalized anxiety disorder

The group consisted of 27 participants, mostly women (81.5%), with an average age of 39.11 ± 13.53 years; 51.9% were single; 55.6% had finished high school studies and had 12.56 ± 2.93 years of study; and 79.8% with some work or academic activity.

The correlation of HAM-A scale scores and those of defense mechanisms was significant for reaction formation (*r* = 0.414 and *P* = 0.32), projection (*r *= 0.477 and *P* = 0.32), excision (*r* = 0.545, *P* = 0.003), and somatization (*r* = 0.573 and *P* = 0.002) in this group. In the multiple regression analysis, only excision (*β *= 0.545, *t* = 3.25, and *P *= 0.003) showed its participation in the intensity of anxiety. The correlation of the HAM-D scale scores and those of the different defense mechanisms was significant for reaction formation (*r* = 0.386 and *P* = 0.047), projection (*r *= 0.507 and *P* = 0.007), displacement (*r* = 0.470 and *P* = 0.013), excision (*r *= 0.493 and *P* = 0.009), somatization (*r *= 0.522 and *P* = 0.006), and sublimation (*r *= 0.40 and *P* = 0.05).

However, in the multiple regression analysis, only displacement (*β *= 0.373, *t* = 2.24, and *P *= 0.034) and excision (*β *= 0.404, *t* = 2.43, and *P *= 0.23) showed their influence on depressive symptomatology. The correlation of the BDI scores and those of defense mechanisms was significant for pseudo-altruism (*r *= 0.457 and *P* = 0.016), projection (*r *= 0.637 and *P* = 0.000), acting out (*r *= 0.428 and *P* = 0.026), fantasy (*r *= 0.410 and *P* = 0.034), displacement (*r *= 0.502 and *P* = 0.008), splitting (*r *= 0.503 and *P* = 0.008), rationalization (*r *= 0.410 and *P* = 0.034), and somatization (*r *= 0.585 and *P* = 0.002). In the multiple regression analysis, the two defense mechanisms that influenced the intensity of depression were autistic fantasy (*β *= 0.379, *t* = 2.53, and *P* = 0.019) and somatization (*β *= 0.555, *t* = 3.70, and *P* = 0.001). Finally, the defense mechanisms used by the participants of the study are given in Table 3.

**Table 3:** Defense mechanisms by the diagnostic groups (DSQ-40). DSQ-40, 40-item Defense Style Questionnaire; MDD, major depressive disorder; PD, panic disorder; GAD, generalized anxiety disorder

	MDD	PD	GAD
n	27	27	27
Sublimation	5.59 ± 2.39	4.94 ± 2.63	4.68 ± 1.88
Humor	4.59 ± 2.19	5.09 ± 1.81	5.75 ± 1.83
Anticipation	5.92 ± 2.36	5.25 ± 2.64	5.51 ± 1.98
Suppression	4.81 ± 2.73	5.20 ± 1.75	4.29 ± 1.87
Annulment	5.11 ± 2.49	4.81 ± 2.02	4.22 ± 1.72
Pseudo-altruism	5.75 ± 2.09	4.09 ± 2.27	3.98 ± 2.19
Idealization	4.16 ± 2.36	3.55 ± 2.10	3.75 ± 2.33
Reactive formation	4.70 ± 1.96	4.33 ± 2.08	4.09 ± 2.16
Projection	4.75 ± 2.16	3.05 ± 2.06	3.59 ± 2.34
Passive aggressiveness	4.25 ± 2.30	3.72 ± 2.18	4.11 ± 1.88
Acting out	6.11 ± 2.71	4.63 ± 2.53	4.42 ± 2.76
Isolation	4.79 ± 2.58	4.18 ± 2.83	3.51 ± 2.23
Devaluation	4.79 ± 2.24	5.03 ± 1.93	4.35 ± 1.65
Fantasy	5.07 ± 2.78	4.35 ± 2.81	4.70 ± 2.59
Denial	4.01 ± 2.39	3.79 ± 2.47	3.98 ± 2.62
Displacement	5.14 ± 2.18	4.05 ± 2.32	4.01 ± 2.45
Dissociation	3.94 ± 1.96	3.48 ± 1.53	3.09 ± 1.48
Excision	5.16 ± 3.01	4.64 ± 2.31	4.31 ± 2.37
Rationalization	6.25 ± 2.18	5.22 ± 2.14	5.64 ± 2.13
Somatization	6.18 ± 2.30	4.85 ± 2.60	4.48 ± 2.53

## Discussion

According to the results obtained in this study, some relationships between the study variables were determined. First, most of the participants were women, a situation that could be in line with previous findings in the psychiatric literature, where it is described that both depressive and anxious disorders are more frequent in this population, at least in epidemiological terms, and in the Americans [4,14]. In the background, and concerning the rest of the sociodemographic variables, it is again identified that the majority of the participants were single, who, in turn, had a medium-high academic degree, a situation that partially reflects the social situation of our country [15].

Regarding the psychometric instruments, it is noteworthy that the mean of the HAM-D score falls in a value that would represent a severe symptom intensity, while both the BDI and HAM-A fall into moderate grades. These results are also striking as they reflect the symptomatic severity of the participants in this study, which, as just mentioned, stands out for not falling to mild levels. This situation could also be interpreted as a bias as the study collaborators are assistants to the services of a tertiary care hospital, so they could represent a minority of the national population that also presents the comorbidities of interest in the study. Likewise, reaction formation, projection, and somatization influenced the score of depressive symptoms on these scales, and, in particular, an important association with the rationalization mechanism was identified. Regarding personality disorders, again some findings are in line with existing knowledge, that is, participants with cluster B personality disorders were predominantly identified, particularly those with a borderline personality disorder. However, no significant differences were found between individuals with MDD, PD, and GAD (and their scales) with the frequencies of personality disorders. This is noteworthy, as the medical literature has established the relationship between borderline traits and depressive comorbidities [16]. It is possible that these differences were not significant in this population due to the size of the sample, and perhaps with a greater number of participants, these could become noticeable.

Regarding the regression analysis, there are circumstances to discuss. For example, fantasy and somatization positively predicted BDI scores in participants with MDD. This finding could suggest that the presence of these defense mechanisms could have a directly proportional relationship with the score of both scales. Similarly, excision predicted the same HAM-A outcome in patients with PD. This discovery is notorious. Defense mechanisms are understood as unconscious phenomena that can dictate the actions of an individual in a specific context. In other words, it would not be surprising if the influence of these defenses was reflected when answering a scale that assesses both symptoms of depression and anxiety. Therefore, it would be important to establish the mechanism of how such defenses impact the evaluation and response of each item of the scales that were used. And, finally, in subjects with GAD, pseudo-altruism, projection, acting out, fantasy, displacement, splitting, rationalization, and somatization both positively and negatively predicted the BDI score. This is also notorious because the essence of this disorder is the difficulty in managing the incessant worry in the face of uncertainty; these defenses, in turn, may represent the multivariate coping style of the population studied [17]. It would be interesting to conduct further studies that address the deeper or even qualitative or psychodynamic relationship of the users of the institution with these conditions and how they could be related to other outcomes of interest in the future.

Although these results are promising, they should be interpreted with caution. This is because Mexico, by being a nation with wide ethnic and cultural diversity, could have mediating social factors that are not contemplated in the design of this project. This point weighs even more, as the participants were recruited from a highly specialized medical institution that concentrates on individuals from various parts of the country. That being said, these variables were not measured; therefore, such an influence could not be measured. This last argument applies, in theory, to every nation, but it is an important detail that must be considered when applying the results of scales that, although standardized and validated, do not necessarily reflect consistent results in people with significant social or even linguistic diversity. Therefore, it is difficult to determine if the findings of this study apply to all Mexicans. An observational or even qualitative study that focuses on these variables could look at whether such a relationship exists in greater depth. However, the academic value of this study lies in the confirmation that these disorders appear to have a certain association and that the use of tools such as those used in this project can be easy to use by Mexican clinicians, facilitating their daily clinical work.

Limitations

While the findings of this study are interesting, the project is not without limitations that should be noted. In principle, the calculation of the sample was conducted in such a way that the results do not necessarily reflect the national population. Second, the attribution bias on the part of the participants is not ruled out, that is, they consciously or unconsciously modified their answers when they knew they were collaborating on a research study. Third, the diagnoses of personality disorders were based on the results of clinimetrics. Thus, these disorders were not ratified through a structured interview. Also, this project is transversal, so the results do not necessarily explain the causality of the phenomena described. In addition, the study does not consider whether the participants had already been diagnosed with a previous psychiatric disorder or if they received any treatment. This last point is especially important, considering that psychotherapeutic interventions could have mediated defensive styles, an issue that was not measured in this project. Finally, the comparison was carried out between three diagnostic groups and there was no control comparison. It would have been interesting to assess whether there are differences regarding the defense mechanisms and traits of participants without diagnoses of MDD, PD, and GAD. These limitations affect the external validity of the study. Due to this, it is important to carry out a prospective, retrospective, or analytical studies that measure these phenomena.

## Conclusions

This research aimed to study, psychometrically measure, and associate personality disorders with defense mechanisms as well as depressive and anxious symptoms in the Mexican population. In this sense, the project's findings ratify those found in the literature and are replicated in these individuals. The defense mechanisms predicted greater symptom severity in the depressive and anxiety scales, particularly with the self-applied ones, a situation that could indirectly reflect the coping style and other elements of the character of the participants, which could be further studied in future research. Also, in this project, both personality disorders and defense mechanisms were measured using standardized psychometric instruments. This is of interest as these constructs are generally assessed either with structured interviews based on diagnostic criteria or with long-term psychodynamic interventions. Having said that, using instruments that can issue data regarding the psychodynamic constructs that it captures in a short period could help guide the diagnosis or course of patients in clinical practice. Similarly, the fact that these results are associated with depressive and anxious symptoms, frequently the most common comorbidities of personality disorders, is consistent with what has already been reported in the literature. However, the aforementioned measures do not replace the known interventions to carry out an integrative psychiatric diagnosis but add value to the weight of these psychometric tools and the impact they can have in daily psychiatric practice. In conclusion, the use of tools such as the PDQ-4 and DSQ-40 in Mexicans is associated with consistent results in this population and with the comorbid diagnosis of depressive and anxious disorders.
